# N-Acetylglucosamine (GlcNAc) Sensing, Utilization, and Functions in *Candida albicans*

**DOI:** 10.3390/jof6030129

**Published:** 2020-08-07

**Authors:** Han Du, Craig L. Ennis, Aaron D. Hernday, Clarissa J. Nobile, Guanghua Huang

**Affiliations:** 1 State Key Laboratory of Genetic Engineering, School of Life Sciences, Fudan University, Shanghai 200438, China; handu@fudan.edu.cn; 2 Institutes of Biomedical Sciences, Fudan University, Shanghai 200438, China; 3 Department of Molecular and Cell Biology, University of California, Merced, Merced, CA 95343, USA; cennis@ucmerced.edu (C.L.E.); ahernday@ucmerced.edu (A.D.H.); cnobile@ucmerced.edu (C.J.N.); 4 Quantitative and Systems Biology Graduate Program, University of California, Merced, Merced, CA 95343, USA

**Keywords:** cell surface receptor, Ngt1, N-acetylglucosamine, GlcNAc, GlcNAc utilization, morphological transitions, virulence, cAMP signaling, *Candida albicans*

## Abstract

The sensing and efficient utilization of environmental nutrients are critical for the survival of microorganisms in environments where nutrients are limited, such as within mammalian hosts. *Candida albicans* is a common member of the human microbiota as well as an opportunistic fungal pathogen. The amide derivative sugar N-acetlyglucosamine (GlcNAc) is an important signaling molecule for *C. albicans* that could be a major nutrient source for this fungus in host settings. In this article, we review progress made over the past two decades on GlcNAc utilization, sensing, and functions in *C. albicans* and its related fungal species. GlcNAc sensing and catabolic pathways have been intensively studied in *C. albicans*. The *C. albicans* protein Ngt1 represents the first identified GlcNAc-specific transporter in eukaryotic organisms. In *C. albicans*, GlcNAc not only induces morphological transitions including the yeast to hyphal transition and the white to opaque phenotypic switch, but it also promotes fungal cell death. The Ras-cAMP/PKA signaling pathway plays critical roles in regulating these processes. Given the importance of GlcNAc sensing and utilization in *C. albicans*, targeting GlcNAc associated pathways and key pathway components could be promising in the development of new antifungal strategies.

## 1. Introduction

The amide derivative sugar N-acetlyglucosamine (GlcNAc) is a component of the peptidoglycan layer of bacterial cell walls and the chitin layer of fungal and parasitic cell walls [1]. It is also found in the extracellular matrix of bacterial biofilms and in the extracellular matrix glycosaminoglycans of animal cells [1]. GlcNAc is a nutrient source for many microorganisms and also plays critical roles in signaling for both bacterial and mammalian cells [1,2,3]. It regulates a variety of biological processes including morphological transitions, virulence, response to oxidative stresses and antifungal agents, and cell death in several fungal species such as *Candida albicans* [1,4,5,6,7], *Candida tropicalis* [8,9,10], *Yarrowia lipolytica* [11], and *Histoplasma capsulatum* [12]. *Candida* species are common members of the human microbiota; however, they are also opportunistic pathogens that can cause superficial and life-threatening infections in humans, especially in immunocompromised individuals [13,14,15,16]. Given the ubiquitous nature of GlcNAc in mammalian and microbial cells, it seems likely that GlcNAc would be abundant in the human host and could serve as a critical signaling molecule that regulates the switch between commensalism and pathogenicity for the *Candida* species. Here, we review the current knowledge of GlcNAc sensing, utilization, and functions in the pathogenic *Candida* species.

## 2. Ngt1, the GlcNAc-Specific Transporter

A comparative proteomic analysis of *C. albicans* plasma membrane localized proteins from yeast and filamentous cells induced by GlcNAc identified the protein Ngt1 (N-acetylglucosamine-specific transporter 1) as a GlcNAc-specific transporter [17], representing the first eukaryotic GlcNAc-specific transporter to be identified. Ngt1 is comprised of 12 transmembrane domains and has structural similarity to the major facilitator superfamily (MFS) membrane transporters. Although both serum and GlcNAc are potent inducers of hyphal development, only GlcNAc was found to induce Ngt1 in *C. albicans* hyphal cells [17]. Interestingly, Ngt1 was also found to be induced by the engulfment of *C. albicans* cells by macrophages [17]. Consistent with the latter finding, transcriptomic analysis showed that the expression of *NGT1* and GlcNAc catabolism-associated genes were upregulated upon macrophage engulfment [18]. Taken together, these observations suggest that GlcNAc sensing and signaling are likely intertwined with the ability of *C. albicans* to interact with and evade the host immune response.

*Saccharomyces cerevisiae,* a distantly related yeast species, does not contain an ortholog of *NGT1* and is unable to utilize GlcNAc as a sole carbon source. Heterologous expression of *C. albicans NGT1* in *S. cerevisiae*, however, can promote GlcNAc uptake, further confirming the role of Ngt1 as a GlcNAc transporter [17,19]. Interestingly, a BLASTP search of *C. albicans* Ngt1 identifies homologs in diverse organisms across fungi, plants, and metazoans, highlighting the importance of GlcNAc sensing and utilization in eukaryotes [12,17,20,21]. For example, the dimorphic Fungus *Histoplasma capsulatum* has two GlcNAc transporters that are homologous to *C. albicans* Ngt1 and are encoded by *NGT1* and *NGT2* [12]. *H. capsulatum* Ngt1 and Ngt2 are required for efficient filamentous growth induced by GlcNAc, suggesting that mechanisms of GlcNAc sensing and transport are conserved in fungi. Interestingly, in the maize plant, *Zea mays*, Nope1 was identified as the first plasma membrane GlcNAc transporter in plants [20]. Ectopic expression of the *NOPE1* gene from *Z. mays* in a *C. albicans ngt1/ngt1* mutant strain was able to restore cell and filamentous growth of the *C. albicans ngt1/ngt1* mutant strain on GlcNAc-containing medium, suggesting that the functions of GlcNAc transporters are conserved across plant and fungal kingdoms [20].

## 3. GlcNAc Catabolism

**Figure 1:**
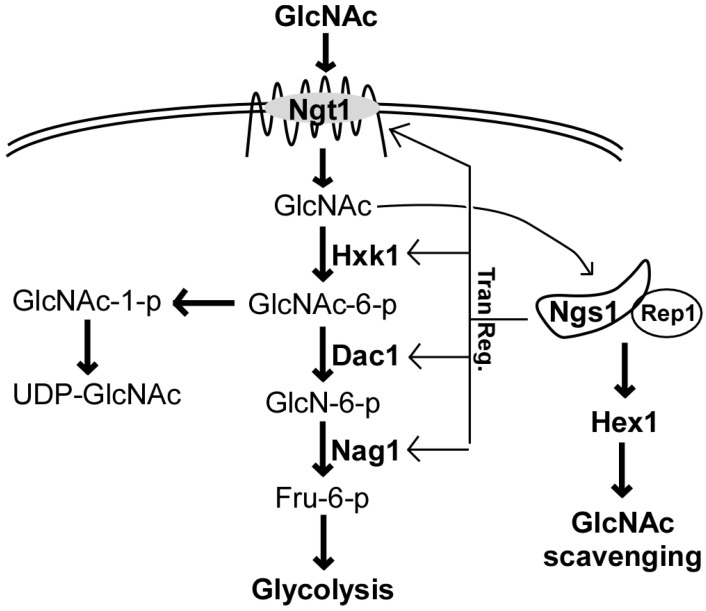
N-acetlyglucosamine (GlcNAc) catabolism in *Candida albicans.* Ngt1, GlcNAc-specific transporter; Hxk1, GlcNAc kinase; Dac1, GlcNAc-6-phosphate deacetylase; Nag1, Glucosamine-6-phosphate deaminase; Ngs1, N-acetyltransferase; Rep1, transcription factor involved in transcription of GlcNAc-inducible genes. GlcNAc is transported into the cytoplasm by the Ngt1 transporter and is catabolized to fructose-6-phosphate by Hxk1, Dac1, and Nag1. Ngs1 can bind to GlcNAc and may function as a putative sensor. Together with the transcription factor, Rep1, Ngs1 induces the expression of GlcNAc-catabolic genes in the presence of GlcNAc.

GlcNAc is catabolized to fructose-6-phosphate in a stepwise manner by GlcNAc kinase (EC 2.7.1.59), GlcNAc-6-phosphate deacetylase (EC 3.5.1.33), and glucosamine-6-phosphate deaminase (EC 3.5.99.6), which are encoded by *HXK1*, *DAC1*, and *NAG1*, respectively (Figure 1) [19,22,23]. The presence of GlcNAc rapidly induces the expression of all three genes that are clustered together in the *C. albicans* genome [19,22]. This clustering of associated genes with related functions is common in microorganisms and may facilitate their coordinated regulation in response to environmental changes.

Recently *NGS1,* which encodes an N-acetyltransferase related to the histone acetyltransferase Gcn5, was identified as a GlcNAc sensor in *C. albicans* [24,25]. Ngs1 is constitutively targeted to the promoters of GlcNAc-inducible genes via the transcription factor Rep1 and is required for their transcriptional activation in response to GlcNAc. Deletion of either *NGS1* or *REP1* in *C. albicans* completely abolishes fungal growth on medium containing GlcNAc as the sole carbon source. GlcNAc also induces the expression of *HEX1*, which encodes a secreted N-acetylglucosaminidase that is required for virulence and for *C. albicans* GlcNAc scavenging during an infection [26,27]. The N-acetylglucosaminidase activity is abolished by mutation of *NGS1* in *C. albicans* cells in the presence of GlcNAc, suggesting that the expression of *HEX1* is controlled in an Ngs1-dependent transcriptional induction manner [24,25] (Figure 1). Another transcription factor, Ron1, was discovered to regulate GlcNAc catabolism and GlcNAc-induced filamentous growth in *C. albicans* [24]. This result is in contrast to the findings in a recent study, where the deletion of *RON1* using the CRISPR/Cas9 method did not affect filamentous growth in response to GlcNAc [28]. However, other phenotypes were indeed observed for the *ron1/ron1* mutant strain in this study that were consistent with its role in GlcNAc catabolism [28]. Moreover, the *ndt80/ndt80 ron1/ron1* double mutant strain exhibited increased filamentation in the presence of GlcNAc. Taken together, these results suggest that Ron1 could function as a negative regulator of filamentation in the absence of Ndt80 [28]. The discrepancies between the two studies could be due to secondary mutations that may have arisen during construction of the different *ron1/ron1* mutant strains used. In *Trichoderma reesei*, a non-pathogenic environmental fungal species distantly related to the *Candida* species, a Ron1 ortholog also controls the expression of a GlcNAc catabolic gene cluster [21], suggesting that the regulation of GlcNAc responsive genes is conserved across the fungal kingdom.

## 4. GlcNAc Regulates Morphological Transitions and Virulence in Fungi

Morphological plasticity is a striking characteristic of pathogenic fungi, and many environmental cues, such as pH, temperature, serum, GlcNAc, and CO_2_, are involved in the regulation of morphological transitions in the *Candida* clade species [5,29,30,31,32,33,34]. *C. albicans*, for example, can transition between several distinct morphological phenotypes [35,36,37,38,39]. Two typical phenotypic switching systems, namely, the yeast–filament transition and white–opaque switching systems, have been extensively studied over the past two decades. Filamentous (hyphal and pseudohyphal) cells are more invasive than yeast-form cells during infections, and the ability to transition between white and opaque cell types is involved in the regulation of both virulence and sexual reproduction in *C. albicans* [35,36,37,38,39]. Morphological changes are often associated with virulence and adaptation to the continuously changing host environment [40]. The earliest observation for a role of GlcNAc in *C. albicans* morphological transitions was reported over forty years ago by Simonetti et al. (1974) [4]. The authors found that GlcNAc induced filamentation in *C. albicans* and hypothesized that GlcNAc is involved in cell wall remodeling during morphological transitions, since chitin (containing GlcNAc), is important to cell wall structure and function [4]. Since this initial discovery, significant progress has been made on elucidating the molecular mechanisms involved in GlcNAc-induced filamentation in *C. albicans* (reviewed by Konopka et al., [1,3]). Filamentation is a key virulence trait in *C. albicans* that is essential for invasive growth in the host. One intriguing study demonstrated that the administration of GlcNAc in a mouse model of oral candidiasis enhanced host tissue damage and increased the fungal burden of *C. albicans* in the mouse [41]. The authors also observed a dose-dependent effect of GlcNAc concentrations on the severity of the infection in the host. This increase in virulence could be the result of enhanced *C. albicans* filamentation.

For the white–opaque phenotypic switching system in *C. albicans*, white and opaque cells differ in colony and cell morphologies, mating competencies, virulence, and transcriptomic profiles [38,40,42,43]. Reversible switching between these two cell types occurs stochastically, yet each cell type is heritable for many generations [33,44,45,46]. The white–opaque switch in *C. albicans* is controlled by the mating type locus (*MTL*) [45]. *MTL* heterozygous cells express the *MTL***a**1/α2 heterodimer, which binds to the promoter of the opaque-specific master regulator *WOR1*, repressing its expression and “locking” cells in the white phase [47,48,49,50]. Our studies demonstrate that GlcNAc can efficiently induce switching to the opaque cell type in *MTL* homozygous (**a**/**a** or α/α) strains, and in a subset of *MTL* heterozygous (**a**/α) clinical isolates [5,51]. Transcription factors Efg1, Rfg1, and Brg1 are involved in the regulation of white–opaque switching in *MTL***a**/α strains [51,52,53]. Since opaque cells are mating-competent, switching to the opaque cell type would facilitate mating and genetic recombination in *C. albicans*. Given that the mammalian gut is a major natural niche for *C. albicans* where commensal bacteria produce GlcNAc and interact with *C. albicans*, this niche could be a conducive environment for phenotypic switching and possibly mating in *C. albicans* cells.

*C. tropicalis* is a member of the *Candida* clade that is closely related to *C. albicans*. Interestingly, unlike in *C. albicans*, GlcNAc represses rather than promotes filamentous growth in *C. tropicalis* [9,10]. However, GlcNAc plays a similar role in the induction of the white-to-opaque transition in *C. tropicalis* [10]. Overall, these results suggest that GlcNAc plays both distinct and conserved roles in the regulation of morphological transitions in these two and perhaps other *Candida* species.

Recent studies have shown that GlcNAc also regulates morphology changes and virulence in other fungal species that have pathogenic potential in humans, such as the environmental fungus *Cryptococcus neoformans* [54] as well as the thermal dimorphic fungi *Histoplasma capsulatum* and *Blastomyces dermatitidis* [12]. The production of melanin by *C. neoformans* is an important virulence factor for this fungal species. Interestingly, GlcNAc affects the cell wall composition, capsule size, and production of melanin in *C. neoformans* [54]. Although the thermal dimorphic fungi are highly evolutionarily diverged from the *Candida* species, these studies highlight the conserved function of GlcNAc as a potent inducer of filamentation across diverse fungal species.

## 5. GlcNAc Can Induce Morphological Transitions Independent of GlcNAc Catabolism

The GlcNAc transporter Ngt1 belongs to the major facilitator superfamily (MFS) and is required for the induction of filamentation in *C. albicans* at low, but not at high, concentrations of GlcNAc [17]. In the closely related species *C. tropicalis*, GlcNAc suppresses filamentous growth, and consistently, Ngt1 is required for this repression [55]. The thermal dimorphic fugus *H. capsulatum* has two homologs of *C. albicans* Ngt1, namely Ngt1 and Ngt2, which are required for GlcNAc-induced filamentation [12]. These findings suggest that the mechanisms of GlcNAc-induced morphological transitions are conserved in fungal species with pathogenic potential.

While GlcNAc catabolism is a key virulence trait in *C. albicans*, it does not appear to be required for GlcNAc-induced filamentation in vitro [19,56,57]. Deletion of the GlcNAc catabolizing enzymes *HXK1*, *NAG1*, or *DAC1* in *C. albicans* led to failures in the cellular utilization of GlcNAc as a carbon source [56]. GlcNAc also inhibited the growth of *nag1/nag1* and *dac1/dac1* mutant strains in the presence of other carbon sources, likely due to the toxic effects of accumulated GlcNAc-6-phosphate in the cells [56]. Moreover, both the *hxk1/hxk1* and *hxk1/hxk1 nag1/nag1 dac1/dac1* triple mutant strains were capable of undergoing filamentation in the presence of GlcNAc [56]. It is also established that the *hxk1/hxk1* mutant strain is capable of switching to the opaque and gray phenotypes in an *MTL*-independent manner [58,59,60]. These results indicate that GlcNAc is involved in the induction of morphological transitions and suggest that GlcNAc-induced morphological transitions are independent of metabolism.

## 6. The Ras1-cAMP/PKA Signaling Pathway Functions in GlcNAc-Induced Filamentation

The conserved Ras1-cAMP/protein kinase A (PKA) signaling pathway is a major regulatory pathway controlling environmental sensing in *C. albicans* [61,62,63]. In addition, this pathway also controls morphological transitions, virulence, and stress responses in *C. albicans* [61,62,63,64]. The major components of this pathway include the small GTPase Ras1, the adenylyl cyclase Cyr1, the cyclic nucleotide phosphodiesterases Pde1 and Pde2, the PKA catalytic subunits Tpk1 and Tpk2, and the regulatory subunit Bcy1 [61]. GlcNAc stimulates filamentous growth and white to opaque cell phenotypic switching in *C. albicans* through activation of the Ras1-cAMP/PKA pathway [5,57,61,65,66]. Inactivation of this pathway by either chemical or genetic perturbation suppresses GlcNAc-induced filamentation and white to opaque switching in *C. albicans* [5,57]. Ras1 is localized to the plasma membranes and/or to the cytoplasm in *C. albicans*, and constitutive activation of Ras1 via a G13V substitution (Ras1V13) leads to an increase in membrane localization and filamentous growth in the presence of GlcNAc [65,67]. The *C. albicans* adenylyl cyclase Cyr1 contains a Ras-binding domain and multiple domains responsible for sensing distinct environmental signals [64]. Deletion of *CYR1* blocks GlcNAc-induced filamentous growth [68], whereas inactivation of the phosphodiesterase *PDE2* enhances filamentous growth [69]. Earlier studies showed that the permeable PKA inhibitors, MyrPKI and H-89, inhibit both GlcNAc- and serum-induced filamentation in a concentration-dependent manner [57]. The PKA regulatory subunit Bcy1 acts as a negative regulator of the cAMP signaling pathway [70,71]. We previously reported that neither the PKA catalytic subunits (Tpk1 and Tpk2) nor the regulatory subunit (Bcy1) are essential for cell survival in *C. albicans* [72]. Consistent with the function of Bcy1 in repressing the cAMP/PKA signaling pathway, deletion of *BCY1* results in enhanced filamentation as well as various pleiotropic phenotypes in GlcNAc-containing medium [73].

Other than the Ras1-cAMP/PKA pathway, additional pathways are also likely to regulate GlcNAc-induced filamentation in *C. albicans* [74,75]. For example, the catabolism of GlcNAc produces ammonia and raises the ambient pH of the environment. This alkalinization favors the growth of filaments in *C. albicans* by activating the Rim101-mediated pH sensing pathway [74,75]. Moreover, this increase in pH has been shown to counter macrophage acidification during engulfment, thereby allowing *C. albicans* to survive within macrophages [76].

## 7. Ras1-cAMP/PKA Signaling Pathway Functions in GlcNAc-Induced White-Opaque Switching

The Ras1-cAMP/PKA pathway also regulates white–opaque switching in *C. albicans* upon environmental changes [33,61]. We found that GlcNAc not only induces white to opaque switching, but that it also stabilizes the opaque cell type at human physiological temperatures (typically, opaque cells would convert en masse to the white cell type under these conditions) [5]. GlcNAc-mediated induction of the opaque cell state occurs primarily through the Ras1-cAMP/PKA pathway [5], while the stability of the opaque state is not dependent on this pathway [77]. Activation of cAMP signaling by constitutive expression of the activated form of Ras1 (Ras1V13), or by mutation of *PDE2*, led to an en masse conversion to the opaque cell type in the presence of GlcNAc. Consistently, strains with deletions of *RAS1* or *CYR1* had remarkably decreased white to opaque switch frequencies compared to the isogenic wildtype strain. Wor1, the master regulator of white–opaque switching in *C. albicans*, is downstream of the Ras1-cAMP/PKA pathway and contains a potential PKA phosphorylation site (T67). Overexpression of *WOR1* in Ras1-cAMP/PKA pathway mutant strains overrides their switching defects and promotes the opaque state, however replacing the T67 residue within the PKA consensus motif with alanine (T67A), which cannot be phosphorylated, impairs the function of Wor1 in regulating white–opaque switching [5]. Overall, these findings suggest that GlcNAc could regulate white–opaque switching through activation of the Ras1-cAMP/PKA pathway and Wor1 phosphorylation [5].

**Figure 2:**
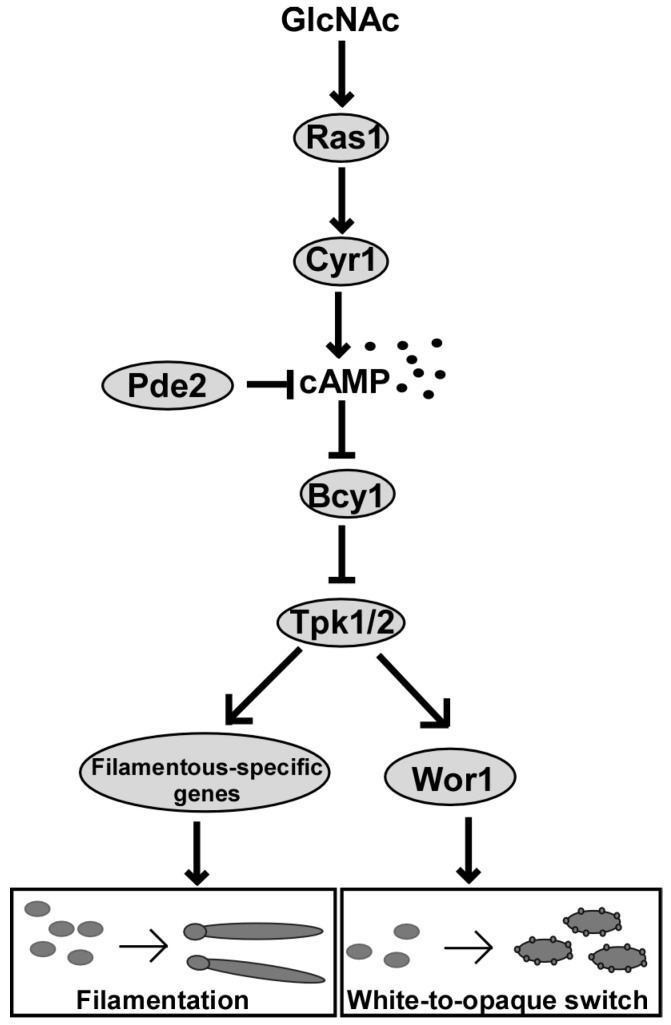
The Ras1-cAMP/PKA signaling pathway regulates GlcNAc-induced filamentation and white–opaque switching in *C. albicans.* GlcNAc may directly activate the small GTPase Ras1. Activated Ras1 stimulates the adenylyl cyclase Cyr1 to produce cAMP, while the cyclic nucleotide phosphodiesterase Pde2 degrades cAMP. In the absence of GlcNAc, the PKA regulatory subunit Bcy1 binds to the catalytic subunits Tpk1 and Tpk2. In the presence of GlcNAc, cAMP binds to Bcy1 and leads to the release and activation of the catalytic subunits that subsequently phosphorylates filamentation-specific or opaque-specific regulators.

The transcription factors Wor2 and Flo8 are also important regulators of white–opaque switching in *C. albicans*, and both are required for inducing switching to the opaque form in medium containing glucose as the sole carbon source [78,79]. However, GlcNAc can induce white to opaque switching and stabilize the opaque phenotype in cells of the *wor2/wor2* and *flo8/flo8* mutant strains, indicating that these two regulators can be bypassed via GlcNAc-induced signaling. Interestingly, constitutive overexpression of the activating form of Ras1 (Ras1V13) hypersensitizes cells of the *wor2/wor2* mutant strain to GlcNAc [78]. Similar to Wor1, Flo8 is also downstream of the Ras1-cAMP/PKA pathway [80]. It plays critical roles in CO_2_-induced filamentation and white to opaque switching in *C. albicans* [79]. CO_2_ and GlcNAc have synergistic effects on inducing the opaque cell state [5]. Taken together, the Ras1-cAMP/PKA pathway functions as a major regulator of GlcNAc-induced filamentation and white–opaque switching in *C. albicans* (Figure 2). Finally, there are also other biological processes that are likely regulated by GlcNAc that have yet to be identified.

## 8. GlcNAc-Induced Cell Death in *C. albicans*

For microorganisms, carbon source limitations frequently occur in nature and the ability to sense and respond to nutrient changes is critical for survival. Under nutrient starvation conditions, *S. cerevisiae* has been shown to enter a G0/G1 phase or to arrest in stationary phase in order to survive [81,82]. The addition of glucose to *S. cerevisiae* cells has been shown to act as a signal for abundant nutrient availability, inducing rapid cell growth [81,82]. However, when incubated in the presence of glucose without other nutrients to support growth, *S. cerevisiae* viability was shown to markedly decrease due to the lack of other nutrients necessary to support growth [81]. Under such conditions, the authors suggest that glucose initiates a new mitotic cell cycle that results in cell death caused by the accumulation of reactive oxygen species (ROS). This glucose-induced cell death in *S. cerevisiae* is dependent on the cAMP signaling pathway and the rate of sugar phosphorylation [81,83]. In general, this phenomenon is called sugar-induced cell death (SICD) and shares characteristics with programmed cell death such as nuclear DNA fragmentation and phosphatidylserine externalization [84].

Interestingly, GlcNAc, but not glucose, induces cell death in *C. albicans* due to the accumulation of ROS and its associated cell damage [6]. The GI tract, a natural niche for *C. albicans*, contains many commensal bacteria that contain peptidoglycans in their cell walls [85,86]. Although GlcNAc levels within the host have not been directly measured, based on the abundance or peptidoglycan producing bacteria residing in the healthy GI tract, it seems likely that GlcNAc would be an abundant carbon source within this niche. A study by Chang et al. (2004) suggested that GlcNAc is a major nutrient carbon source for *Escherichia coli* in the mouse gastrointestinal tract [87]. *C. albicans* may also use GlcNAc rather than glucose, which is scarce in the lower gastrointestinal tract, as a signal for nutrient availability. RNA-Seq analysis demonstrated that GlcNAc treatment increased the expression of ribosomal biogenesis genes in *C. albicans*, suggesting that protein biosynthesis and metabolism are active, and cells are induced to enter the mitotic phase in the presence of GlcNAc [6]. The cAMP signaling and GlcNAc catabolic pathways are involved in GlcNAc-induced cell death. Although deletion of *RAS1* did not affect the efficiency of GlcNAc-induced cell death, inactivation of the adenylyl cyclase-encoding gene *CYR1* significantly delayed cell death in *C. albicans* [6]. Consistent with this finding, activation of the cAMP signaling pathway by constitutive expression of Ras1 (Ras1V13) or by deletion of *PDE2* accelerated cell death in the presence of GlcNAc [6]. As expected, deletion of *NGT1* or genes of the GlcNAc catabolic pathway also delayed GlcNAc-induced cell death in *C. albicans*, suggesting that metabolism of GlcNAc is required for this induction. Moreover, it was found that a *Candida* clade-specific mitochondrial protein, Mcu1, is required for the utilization of GlcNAc in *C. albicans*. In this study, the deletion of *MCU1* resulted in a decrease in susceptibility to GlcNAc and delayed cell death [6]. Furthermore, *C. albicans MCU1* was also found to be essential for GlcNAc utilization and filamentation in several filament-inducing conditions as well as for virulence in a mouse systemic infection model [88]. Given the distinct natural niches for *C. albicans* and *S. cerevisiae*, it is feasible that these two microorganisms have evolved to detect different carbon sources as signals for nutrient abundance. However, the overall responses to these carbon sources (initiation of cell growth or cell death) are similar between these two distantly related species, suggesting that features of this nutrient response mechanism are conserved.

Interestingly, adjacent to *MCU1* is a pseudogene (*ORF19.4984*) that is predicted to encode a putative LysM domain-containing protein in *C. albicans* [88]. Fungal LysM domain-containing proteins often bind to chitin or GlcNAc and function as effectors to suppress plant and insect host immune responses [89,90]. The related *Candida* species, *C. tropicalis*, is widespread in the environment and is known to contain several genes that encode LysM domain-containing proteins [91,92]. These proteins may aid *C. tropicalis* in scavenging for and efficiently utilizing environmental chitin as a nutrient source. As a human commensal, *C. albicans* may not require LysM domain-containing proteins to the same degree as environmental fungi to acquire nutrients in the human host. Taken together, Mcu1 and the putative LysM domain-containing protein may have been functionally associated before *ORF19.4984* became a pseudogene and before *C. albicans* evolved to colonize humans.

## 9. Conclusions and Open Questions

GlcNAc is an abundant carbon source in the environment and within a mammalian host and is utilized as a nutrient and signaling molecule in organisms ranging from bacteria to fungi to humans [1,3]. *C. albicans* can utilize GlcNAc as a carbon source through its GlcNAc catabolic pathway. In addition to being a nutrient source, GlcNAc functions as a signaling molecule that can induce filamentous growth and white to opaque switching in *C. albicans*. Given that these two morphological transitions have been extensively studied in *C. albicans*, this fungal species is an ideal model system to further explore the underlying molecular mechanisms of GlcNAc sensing and utilization. Despite the remarkable advances in our understanding of GlcNAc sensing that have been made over the past two decades, many important questions remain to be answered. For example, given the close phylogenetic relationship between *C. albicans* and *C. tropicalis*, why does GlcNAc have opposite effects on filamentation in these two fungi? The Ras1-cAMP/PKA signaling pathway is a major regulator of GlcNAc-induced morphological transitions and cell death in *C. albicans*. Given that Ras1 can be found at both the membrane and the cytoplasm, does GlcNAc directly bind Ras1 and stimulate its activation? Does this binding event affect Ras1 localization? How does GlcNAc signaling, using the same Ras1-cAMP/PKA signaling pathway, regulate different biological processes and phenotypic outputs such as yeast to hyphal growth, white-to-opaque switching, and cell death? Other than its known role as a transporter, does Ngt1 also function as a sensor to detect GlcNAc? Do Ngt1 and Ras1 coordinate to regulate GlcNAc sensing? Does GlcNAc sensing, utilization, and/or the resulting functional outputs of these processes play roles in commensalism? Lastly, how does *C. albicans* integrate GlcNAc signaling with other environmental signals to respond to changes in the host environment? Mechanistically answering these questions will not only increase our understanding of the survival strategies used by *C. albicans* in the host, but will also likely lead to the identification of novel antifungal targets for therapeutic development.
